# Dynamic data-enabled stratified sampling for trial invitations with application in NHS-Galleri

**DOI:** 10.1177/17407745231167369

**Published:** 2023-04-24

**Authors:** Adam R Brentnall, Chris Mathews, Sandy Beare, Jennifer Ching, Michelle Sleeth, Peter Sasieni

**Affiliations:** 1 Wolfson Institute of Population Health, Centre for Evaluation and Methods, Queen Mary University of London, London, UK; 2 The Cancer Research UK and King’s College London Cancer Prevention Trials Unit, Kings College London, London, UK

**Keywords:** Cancer, equity, invitations, recruitment, screening

## Abstract

**Background:**

Participants of health research studies such as cancer screening trials usually have better health than the target population. Data-enabled recruitment strategies might be used to help minimise healthy volunteer effects on study power and improve equity.

**Methods:**

A computer algorithm was developed to help target trial invitations. It assumes participants are recruited from distinct sites (such as different physical locations or periods in time) that are served by clusters (such as general practitioners in England, or geographical areas), and the population may be split into defined groups (such as age and sex bands). The problem is to decide the number of people to invite from each group, such that all recruitment slots are filled, healthy volunteer effects are accounted for, and equity is achieved through representation in sufficient numbers of all major societal and ethnic groups. A linear programme was formulated for this problem.

**Results:**

The optimisation problem was solved dynamically for invitations to the NHS-Galleri trial (ISRCTN91431511). This multi-cancer screening trial aimed to recruit 140,000 participants from areas in England over 10 months. Public data sources were used for objective function weights, and constraints. Invitations were sent by sampling according to lists generated by the algorithm. To help achieve equity the algorithm tilts the invitation sampling distribution towards groups that are less likely to join. To mitigate healthy volunteer effects, it requires a minimum expected event rate of the primary outcome in the trial.

**Conclusion:**

Our invitation algorithm is a novel data-enabled approach to recruitment that is designed to address healthy volunteer effects and inequity in health research studies. It could be adapted for use in other trials or research studies.

## Background

Participants in clinical trials are usually healthier than the target population. This so-called healthy volunteer effect has been observed in most cancer screening trials done to date. For example, in the Prostate, Lung, Colorectal and Ovarian (PLCO) cancer screening trial, participants in the control arm had less than half the rate of mortality than the general population^1^; a similar effect was seen in the European Randomised study of Screening for Prostate Cancer (ERSPC)^2^; and mortality was lower in participants than those who did not join the lung-screening NELSON (Nederlands–Leuvens Longkanker Screenings Onderzoek) trial.^3^ Healthy volunteer effects have also been observed in cohort studies including the European Prospective Investigation into Cancer and Nutrition (EPIC)^4^ and UK Biobank.^5^ A parallel issue is that participants recruited to such research studies are usually much less diverse than the target population. For example, ethnic minorities were under-represented in the PLCO cancer screening trial, despite efforts^6^; those who joined UK Biobank were disproportionally from less-deprived areas.^5^

It is important to try to address healthy volunteer effects and representation of the target population at the design stage of research studies for several reasons. First, unless accounted for the study will be underpowered. Second, lack of representation risks generalisability. Third, seeking to limit healthy volunteer effects and trying to ensure all groups of society are represented in adequate numbers is important for moral reasons. There is an imperative to reduce health inequalities in all areas, including representing those who most likely to have ill health in research.^7^

In this article, we outline a dynamic data-enabled method for inviting people to join a trial. It is designed to help address healthy volunteer effects and improve representation. The approach was developed for the NHS-Galleri trial (ISRCTN91431511).^8^ This trial is being run to see how well a multi-cancer early detection test (Galleri® test) works in the National Health Service (NHS) in England.^9^ The trial aim is to evaluate if the test (alongside standard screening) finds cancer earlier and thereby prevents stage III and IV cancers in people who do not have symptoms of cancer.

Clinical and demographic factors were monitored during recruitment to try to ensure that: (1) the participants at entry would be representative of the population of England aged 50–77 years; and (2) the incidence of advanced cancer in the control arm within 3 years of enrolment would be at least as great as the average among the population of England age 50–77 years. By ‘representative’, we mean participants from all areas of deprivation and all major ethnic groups should be included in reasonable numbers. We do not mean that the proportion from each group should exactly mirror that of the population as a whole. Indeed, we would prefer to over-recruit from more deprived groups and ethnic minorities, because people in these groups are usually substantially under-represented in clinical trials and will have poorer health outcomes because of the social determinants of health.^7^ In other words, the recruitment strategy aimed for equity rather than equality. We also note that if all major ethnic and deprivation groups are represented in the study sample then marginal measures may be calibrated to different populations through standardisation methods that differentially weight data from participants. Under-sampling uncommon groups will decrease the precision of standardised estimates much more than under-sampling common groups.

One recruitment strategy is to allow anyone eligible to be able to join. This has consistently been shown to suffer from healthy volunteer effects. Another approach is to require that participants receive an invitation before joining. This approach was used in the United Kingdom Collaborative Trial of Ovarian Cancer Screening (UKCTOCS).^10^ Women were randomly invited from population registers. The trial invited 1,243,282 women to recruit 205,090 (uptake 16.5%).^11^ Unfortunately, on average those who joined the study were less deprived than the wider population, and mortality in the trial was substantially less than the wider population.^10^ The trial leaders had to extend the duration of screening and follow-up to achieve a sufficient number of events in the control arm for their primary analysis.^10^

An alternative to random invitation is stratified sampling. This was used in the NHS-Galleri trial. The vast majority of participants were invited to attend a mobile clinical unit for blood sampling. Invitations were sent to patients registered with a General Practitioner (GP) located in a geographical circumference around the clinical unit or site in accordance with the relevant permissions and approvals. A dynamic computer programme was used to decide which groups of people to invite through NHS DigiTrials, to ensure adequate representation in participants across demographic and clinical factors, enrich for advanced cancer in the control arm and account for likely healthy volunteer bias. In addition to the central approach, there was also targeted GP search invitations, and targeted open enrolment of interested individuals who learned about the trial from specific recruitment efforts in selected communities.^12^ Local media campaigns were coordinated with site openings. Public and patient involvement in the recruitment of participants included the design of participant information materials. Further work is ongoing focussing on behavioural science relating to acceptability and informed decision-making when considering participation in screening using tests for multiple cancer types.^13^

In the rest of this article, we report the algorithm that was developed and used for most of the invitations to NHS-Galleri and describe how its parameters were set. The algorithm is sufficiently generic that it might also be useful beyond this trial for other research studies.

## Methods

### Model

**Figure 1.**
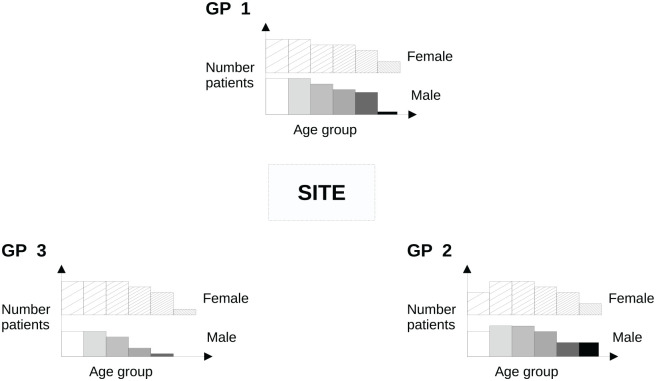
Schematic of the invitation model in an example where (for simplicity) the site is served by three GP practices (clusters). There is an age/sex distribution of people to potentially invite within each cluster. This is illustrated using a histogram, where the solid blocks represent the male population and the hatched blocks the female population, with increasing age from left to right and from lighter to darker shades. The problem is to determine the number to invite to attend appointments at the site from each age/sex band (group) and GP (cluster).

Our model requires patients to be recruited from different physical locations or periods in time, which we call *sites*. In NHS-Galleri, a site was a location where blood was donated in a mobile clinic. The sites are served by *clusters* of potential participants. In NHS-Galleri, these were patients registered at GPs, in other studies they might be people resident in a geographical area. Each cluster may be further divided into defined *groups*, such as age-and-sex bands. Figure 1 illustrates that the cluster size (number of people registered at each GP) may vary overall, and by age and sex.

Our invitation model is dynamic because invitations are sent in sequential waves within each site. This enables feedback on uptake, which may be used to help plan subsequent waves of invitations. It also provides flexibility if the total capacity at a site changes. For example, a site may be forced to reduce the number of slots available due to logistical issues; or additional capacity is made available.

The invitation problem is to determine the number of people to invite from each group within each cluster serving a single site in each wave, so that the study sample is likely to be adequately powered to meet the trial objective; representative of the population in the sense described above; and all slots available for recruitment to the study are filled. We next describe a mathematical model for this problem. For ease of exposition, in the rest of the article, the model groups are referred to as age/sex groups, and clusters as GPs.

### Optimization problem

The optimization problem is set up and solved separately for each site. For each site, there are j=1,…,J GPs, k=1,…,K age-and-sex groups, and i=1,2,…,W invitation waves. J and W may vary between sites, but K is constant. The problem is to determine the proportion of patients that are invited, $x_{ijk}$ ; given $n_{jk}$ , the maximum number who could be invited from GP j , age/sex-band k . We assume expected uptake $u_{jk}$ from GP j and age/sex-band k is known; also the relative ‘cost’ $c_{ijk}$ of recruiting a patient from GP j in age/sex-band k during wave i . Note that the ‘cost’ here is not financial but a way to express our priorities in terms of who to recruit. For each wave i=1,2,… , the objective is to minimise the expected cost of bookings

$$\mathrm{mi}n_{x_{i}}\sum_{j=1}^{J}\sum_{k=1}^{K}c_{ijk}n_{jk}u_{jk}x_{ijk}$$

by solving for $x_{i}=(x_{i11},\ldots ,x_{ijk})$ , subject to the following constraints.

1. The decision variable $x_{ijk}$ is a real number between 0 and 1 (i.e. proportion). For j=1,…,J and k=1,…,K and i=1,2,…

$$0\leq x_{ijk}\leq 1;x_{ijk}\in R.$$

2. No more than 100% of patients in an age-and-sex group may be invited through the waves i=1,2,… . For j=1,…,J and k=1,…,K

$$x_{ijk}+a_{ijk}\leq 1$$

where $a_{ijk}$ is the known proportion of patients already invited from GP j and age-group k during earlier waves, and $a_{1jk}=0$ for all j,k .

3. The expected number of people who book appointments $B_{i}$ following wave i is fixed:

$$\sum_{j}\sum_{k}n_{jk}u_{jk}x_{ijk}=B_{i}.$$

This constraint effectively controls the number of invitations sent given $n_{jk}$ and $u_{jk}$ . $B_{i}$ will usually be less than the total number of slots available T .

4. The proportion of invitations sent to each GP in each wave is less than a chosen $G_{j}$ . For each wave, i=1,2,… and GP j=1,…,J

$$\sum_{k}x_{ijk}n_{jk}\leq G_{j}\sum_{k}n_{jk}.$$

This is used to avoid GPs being potentially overburdened with inquiries about the trial if, for example, everyone in their practice receives an invitation on the same day.

5. A minimum bound is achieved on the expected proportion of patients who book (of the total) from each age/sex group in wave i . For k=1,…,K

$$\sum_{j}n_{jk}u_{jk}x_{ijk}\geq d_{ik}$$

where $d_{ik}$ is the minimum number of expected bookings in each age/sex band k at wave i . This constraint is useful if one wishes to avoid inviting, for example, only older people.

6. The expected proportion of men who book is S . Suppose that the groups by sex are further stratified, such as by age. Let the k index be ordered by sex, so that k=1,…,K/2 are male and k=K/2+1,…,K are female. Then, for k=1,…,K/2 , we require

$$n_{jk}u_{jk}x_{ijk}(1-S)/S-$$

$$n_{j,k+K/2}u_{j,k+K/2}x_{ij,k+K/2}=0$$

In practice, uptake rates often differ by age and sex, and one may need to invite more men to achieve parity in bookings by age/sex.

7. The expected number of events in those who book is greater than a bound:

$$\sum_{j}\sum_{k}e_{jk}n_{jk}u_{jk}x_{ijk}\geq E_{i}$$

where $e_{jk}$ is the expected event rate of the primary outcome from GP j and age-group k , and $E_{i}$ is the chosen bound.

A summary of all the parameters defined above is in Table 1. The mathematical formulation may be solved using standard methods, such as a simplex algorithm.^14^

**Table 1.** Definition of parameters in the linear programme, and how they were applied in NHS-Galleri.

	Parameter	How used in NHS-Galleri
(a) Capacity		
T	Target total bookings	Defined based on site capacity
$t_{jk}$	Number of slots already booked cluster j , group k	Number of bookings by age/sex/GP, where total is denoted $t_{++}$ , and total by age/sex-group $t_{+k}$ . When no invitations have been sent $t_{jk}=0$
(b) Decision variable		
$x_{ijk}$	Proportion to invite from cluster j , group k during iteration i	Usually up to i=1,2,3,4 waves of invites; Cluster j is GP serving the site; Group k is age-sex group (by sex age 50–75 in 5-year groups; 75–77 years)
(c) Objective function parameters		
$c_{ijk}$	Cost of including an individual from cluster j and group k at iteration i	Cost of inviting a registered patient from GP j in age/sex-band k at iteration i , where $c_{ijk}=e_{jk}+\{1+\max(e_{jk})\}{\tilde{c}}_{j}$
${\tilde{c}}_{j}$	Used in the definition of $c_{jk}$ ; rank order preference of cluster j	Preferences were defined using public data and rules based on location, ethnicity and deprivation of each practice in the vicinity of the site
$n_{jk}$	Maximum number who could be invited from cluster j , group k	Maximum number who could be invited from GP j , age/sex-band k , from public data
$u_{jk}$	Expected uptake (proportion) from cluster j , group k	Expected uptake from GP j, age/sex-band k . Based on assumption or model
(d) Constraints		
$a_{ijk}$	Proportion already invited from cluster j and group k at iterations i	Known proportion of patients already invited from GP j and age-group k at earlier waves. Note that $a_{1jk}=0$ for all j,k , and for iteration i the algorithm uses $a_{\mathrm{ljk}}$ for l=1,…,i−1 ) only. In the trial, we had feedback data from NHS DigiTrials for this
$B_{i}$	Target number to book in wave i	$B_{1}=T/2$ ; $B_{2}=(T-t_{++})/2$ ; $B_{3}=1.1*(T-t_{++})$ ; if required $B_{4}=(T-t_{++})$
$D_{k}$	Minimum bound on expected proportion bookings (of total bookings) by group k	There were six age groups (50–75 by 5 years, 75–77), with minimum, respectively, 7%, 8%, 10%, 12%, 12%, 6%.
$d_{ik}$	Lower bound on number expected to book in group k during wave i . Defined to achieve $D_{k}$ overall taking into account $t_{jk}$	First wave $d_{1k}=D_{k}B_{1}$ . Subsequently, for i=2,3,4 we used the number booked $t_{+k}$ by age/sex group k and overall $t_{++}$ to date, setting $d_{ik}=\max(0,D_{k}(t_{++}+B_{i})-t_{+k})$
$e_{jk}$	Expected event rate of the primary outcome in cluster j group k	Expected advanced cancer incidence by age/sex group k for GP j Based on advanced cancer incidence by age/sex/deprivation from cancer registries, and public data on deprivation by GP
E	Target minimum expected event rate from all bookings	20% higher than the expected advanced cancer rate in the general population
$E_{i}$	Target minimum expected number of events from wave i in order to achieve E	$E_{i}=E(t_{++}+B_{i})-\sum_{j}\sum_{k}e_{jk}t_{jk}$ .
$G_{j}$	Maximum proportion of a cluster that may be invited at each wave i	$G_{j}=0.5$ for all GPs, to guard against a potential flood of calls to the surgery about the trial
S	Target proportion of male versus female expected to book	S=0.5 for parity

GP: general practitioner; NHS: National Health Service.

## Results

We next describe how the algorithm parameters were chosen for NHS-Galleri.

### Algorithm parameters

#### Cost weights

The most important parameter is $c_{ijk}$ , the relative cost of inviting patients from GP j in age/sex-band k in wave i . In the NHS-Galleri trial, we set $c_{ijk}=c_{jk}$ , so that cost did not depend on wave i . We used two criteria to define the costs. First, we wished to prioritise invitations to groups with a higher expected primary outcome event rate ( $e_{jk}$ , annual advanced cancer incidence per thousand patients). Second, we wanted to order GPs by preference, so that if feasible everyone from the first grouping would be invited before the second grouping and so on. Let ${\tilde{c}}_{j}$ be the GP j rank (first preference $\tilde{c}=1$ , etc). Cost was taken to be:

(1) $$c_{jk}=e_{jk}^{-1}+\{1+\mathrm{ma}x_{k}(e_{jk}^{-1})\}{\tilde{c}}_{j}.$$

The first term on the right-hand side gives a higher cost to invitations sent to patients with lower event rates. The second term on the right-hand side is used so that the cost of inviting any patient from the highest preference group of GPs is less than any patient GPs with a lower preference. Therefore, unless the constraints are broken, the optimal solution will be to invite everyone in the highest preference group of GPs before moving to the next preference group. Likewise, the cost for inviting a patient from the second priority group is less than any patient in the third, fourth or lower preference practices. The first term on the right-hand side of equation (1) means that within GPs of the same rank, invitations to patients with the highest advanced cancer rate $e_{jk}$ will have the lowest cost and minimise the objective function. Practically, this objective function rewards inviting older patients from GPs in more deprived areas within each ranking group, since higher advanced cancer rate is linked to older age and more deprivation.

The preference ranking $\left({\tilde{c}}_{j}\right)$ used in equation (1) provides a way to incorporate other factors affecting the perceived utility of a GP than just the expected event rate. For instance, in the NHS-Galleri trial, this was set based on the proximity of GPs to the mobile units serving the site, deprivation and ethnicity mix. More generally, this term may be used to handle factors at the GP rather than the individual level. The ranking is also a way to override automated priority lists based on other factors including local knowledge and to prioritise invitations to areas with greater ethnic diversity even if they are more distant from the site.

Finally, we note that in NHS-Galleri invitation weighting of deprivation and ethnicity information was derived at the *cluster* (GP) level in our model; and age and sex were controlled at the *group* level in our model. This was due to constraints in how participants could be selected for an invitation. The choice between cluster and group factors in future studies will also be dictated by the level of stratification that is feasible.

#### Event rates and uptake

We modelled expected advanced cancer incidence $e_{jk}$ using available data from the cancer registry in England (NCRAS)^15^ to tabulate cancer diagnoses by site, age, sex, stage and derived quintile of the index of multiple deprivation (IMD). These data were used to estimate advanced cancer rate by age/sex/GP by combining them with information on the distribution of IMD quintile in patients registered at each GP.

For uptake, initially we had no data and set $u_{jk}=0.1$ , the same for all GPs and age/sex groups, based on subjective judgement. Once data began to accrue on actual uptake we used observed uptake in the trial by age/sex $\left(u_{jk}=u_{k}\right)$ , so that the number of invitations were adjusted based on age- and sex-specific uptake observed in the trial. When there were sufficient data to explore variation between GPs, we fitted a regression model to take into account an observed strong relationship between uptake and deprivation, and bowel-screening uptake. Initially, we used bowel cancer screening uptake because we thought it would be a good surrogate for engagement by people aged 50–77 years in preventive medicine. We continued to use it because it was a good predictor of uptake. Denoting normalised deprivation summary of GP j by $z_{j1}$ and bowel-screening uptake $z_{j2}$ , our model was of form

$$\mathrm{logit}(u_{jk})=\beta_{k}+\gamma_{1}z_{j1}+\gamma_{2}z_{j2}+\gamma_{3}z_{j2}^{2},$$

where logit(.) is the logistic function, and $\left(\beta_{1},\ldots ,\beta_{K},\gamma_{1},\gamma_{2},\gamma_{3}\right)$ are the unknown parameters. The parameters were fitted by the maximum likelihood using data on the number of bookings and invitations sent for each age/sex group k=1,2,…,12 and GP j=1,…,J . Variable selection was based on exploratory data analysis and iterative model fitting and inspection. The model was used to estimate $u_{jk}$ for each GP j in the country and age/sex group k , and was periodically updated as the trial progressed.

#### Invitation process control

The first invitation process parameter is the target number to book in each wave $\left(B_{i}\right)$ . One approach would be to try to fill all available slots T immediately, ie. $B_{1}=T$ . However, in this case, there is a risk that too many invitations are sent, with implications for postage costs and disappointment of potential participants. On the other hand, if it is low (say $B_{1}=T/10$ ), not all slots will be filled. We initially used $B_{1}=T/2$ , or 50% of capacity assuming projected uptake in the first wave, $B_{2}$ to be 50% of the remaining capacity after the first wave bookings, that is, $B_{2}=(T-t_{++})/2$ ; and $B_{3}$ to be 110% of the remaining capacity. The latter was more than 100% to ensure all slots are filled, accepting that some sites will have people who are unable to join although they wish to.

The second control parameter is the maximum proportion of a GP list that may be invited at each wave. This was arbitrarily set as $G_{j}=0.5$ for all GPs j .

The third control parameter is the minimum number expected to be book in each age-group during each wave $\left(d_{ik}\right)$ . In the first wave, we defined $d_{1k}=D_{k}B_{1}$ , where $D_{k}$ is the parameter to control the proportion in each age/sex group. In subsequent waves i=2,3,… , we know the number of bookings $t_{+k}$ by age group and overall $t_{++}$ to date, and to maintain a minimum $D_{k}$ overall, we set $d_{ik}=\max\{0,D_{k}(t_{++}+B_{i})-t_{+k}\}$ .

The final control parameter is the minimum expected number of events $\left(E_{i}\right)$ achieved in the bookings at iteration i , to meet an overall expected event rate from all round greater than E . This is the primary way to control the extent to which bookings are tilted towards a higher-risk group to help mitigate likely healthy volunteer bias. We set E to be at least 20% greater than the expected event rate for the general population at each site. Then $E_{i}=E(t_{++}+B_{i})-\sum_{j}\sum_{k}e_{jk}t_{jk}$ , so that a lower risk group is permitted to be invited if those booked to date are already high risk. Practically, we view the main value of E as a being parsimonious way to tilt the sample invited towards higher deprivation and/or older age, rather than in guaranteeing a certain event rate.

### Computer algorithm

In our implementation of the algorithm, the parameters in Table 1 were organised into four input CSV files (Table 3). The input files were generated using scripts written in the statistical computing software R. The linear programme was solved using a programme written in Python 3, using the cvxopt library.^14,16^ The algorithm writes a CSV file with the number of people to invite for each wave by age, sex and GP (Table 3). A demonstration example is provided with open source code.^17^

**Table 2.** Public data sources used to help guide invitations in NHS-Galleri

Description	Source	Where used
GP practice postcode	NHS digital^18^	To identify GPs close to site. Lists name, address, postcode and identifying codes for all GP Practices in England and Wales
Postcode directory	Office for National Statistics^19^	Longitude and latitude of postcodes for the site and GPs. To identify GPs in the vicinity of planned units
Ethnicity by lower layer super output area (LSOA)	England and Wales Census 2011^20^	To help prioritise GPs that are distant from the site but serve populations with a wider ethnicity mix (i.e. helped to inform ${\tilde{c}}_{j}$ )
Patients registered at a practice by age/sex	NHS Digital^18^	To determine the maximum number to invite $\left(n_{jk}\right)$
National opt-out statistics	NHS Digital^21^	Statistics on the number of patients registered with GPs who have opted out of having their data used for purposes beyond individual care: such patients were not invited to the trial. Information on opt-outs at a GP level were used to adjust the expected list size $\left(n_{jk}\right)$ for invitations, and to subsequently inflate the number requested, since opt-outs could only be removed from invitations requested in real-time after the request was made
Deprivation score (IMD 2019)	Fingertips (indicator id 93553)^22^	In model for estimated uptake to NHS-Galleri (covariate in model for $u_{jk}$ )
Persons, 60–74 years, screened for bowel cancer within 6 months of invitation (Uptake, %)	Fingertips (indicator id 92601)^22^	In model for estimated uptake to NHS-Galleri (covariate in model for $u_{jk}$ )
Cancer registry	NCRAS	$e_{jk}$ , $c_{jk}$

GP: general practitioner; IMD: index of multiple deprivation; NHS: National Health Service; LSOA: Lower layer Super Output Area; NCRAS: National Cancer Registration and Analysis Service.

**Table 3.** Organisation of algorithm input and output from each wave of invitations

Description	Data	What used for	Change each wave?
(a) Input files (CSV)			
1. Size of GP lists, number of invitations and bookings to date	GP ID; preference rank $\left({\tilde{c}}_{j}\right)$ ; estimated number eligible to invite $\left(n_{jk}\right)$ ; proportion previously invited by age/sex $\left(a_{jk}/n_{jk}\right)$ ; number booked to date $\left(t_{jk}\right)$	To define the invitation problem	Yes
2. Objective function costs and expected event rates	GP ID; age/sex index (k) ; Cost $\left(c_{jk}\right)$ ; Expected event rate $\left(e_{jk}\right)$	To define the objective function, and parameters used in some constraints	No
3. Expected uptake	GP ID; Expected uptake by age/sex k	Model estimates (or otherwise) to obtain the expected uptake from the invitation schedule based on $x_{jk}$	No, unless uptake model updated
4. Expected opt-outs	GP ID; multiplication factor by which to inflate the number of invitations requested from NHS Digital	Needed to account for national data opt-outs by GP, which for governance reasons are removed by NHS Digital *after* individual patients have been randomly selected from GP lists, i.e. It is not possible to request exact number of invites to be sent	No, unless updated by NHS Digital
(b) Configuration parameters (TXT)			
1. Number slots	Based on site capacity	To plan the number of slots to fill	No, unless capacity changes
2. Invitation round	1, 2, 3 or 4	To plan the number of invites to send this round	Yes
3. Uptake adjustment	Default is 1.0; 2.0 would double the predicted uptake $u_{jk}$	When data indicates poor calibration of the uptake assumption	Yes if required
(c) Output file (CSV)			
1. NHS Digital invitation request	GP ID, age band, sex, and number of invitations requested	Formatted in a CSV file for use by NHS Digital to select people for invitations	Yes

GP: general practitioner; NHS: National Health Service.

## Conclusion

We have described a novel data-enabled algorithm to help overcome healthy volunteer effects and improve equity when recruiting to large trials or cohorts. In NHS-Galleri, the method was intended to tilt the invitation sampling distribution towards more deprived groups, and those with a higher expected event rate of the primary outcome in the trial. The approach is unlikely to eliminate all healthy volunteer effects. However, it tries to mitigate the impact of healthy volunteer bias by guarding against potential loss of power, as well as increasing representation in the trial from societal groups who are often not well represented.

The successful use of this algorithm at scale has been demonstrated by rapid recruitment to NHS-Galleri. Approximately 1.5 million people from the general population of England were invited and 140,000 of those were enrolled in under 11 months.^12^ Our method might be used for other research studies. The most direct application would be in other screening trials run through NHS DigiTrials. So that other trial units can build on our methodology, demonstration code has been made available.^17^

There are several considerations for future use of this methodology. The first consideration is the primary endpoint. In NHS-Galleri, the primary endpoint was advanced cancer incidence. There will be different considerations for other outcomes such as cancer-specific mortality. For example, in UKCTOCS, healthy volunteer effects had a greater impact on mortality than on cancer incidence.^10^ One reason for this is the eligibility criteria. These precluded people with cancer from joining the trial, so that those who joined would not have the same cancer-specific mortality rates as the general population in the short to medium term. A second consideration is the choice of variables used to tilt the sample to a higher-risk group. In this example, age, sex and deprivation were the key variables, but a different approach might be needed depending on the trial endpoint. A third consideration is achieving adequate representation of the target population. Age, sex, deprivation and ethnicity are likely to remain important for equity considerations, but there might be other factors that are important to take into consideration. Finally, the choice of variables used in the model will depend on data availability. For example, if data on body mass index were available at a group or cluster level, then it could contribute to this data-driven approach.

Strengths of our method include that it uses the invitation process to adjust recruitment according to pre-determined factors, and a data-enabled strategy to address important problems related to equity and healthy volunteer effects that have affected many research studies. Data on the effectiveness of our strategy will be presented elsewhere.

A limitation of this approach is that the method is based on the site, cluster, group model, which may not translate to all settings. Another limitation is inclusion/exclusion criteria. The example had inclusive entry criteria, but if the trial needs to be more selective then the approach might be more difficult to apply. The methods also rely on several flows of data, which may be a practical impediment to implementation in other settings outside of NHS DigiTrials. One might also be concerned if the trial successfully over-recruits from target groups who may not usually take up cancer screening, and whether this could affect how health policymakers interpret the results of the trial. However, the goals of recruiting to a trial to evaluate efficacy are usually different from those when evaluating the effectiveness of a proven intervention. Subsequent larger-scale pilots and analyses are usually needed to evaluate and help plan implementation.^23^

In conclusion, healthy volunteer effects and adequate representation have been identified as a problem for many years^24^ but arguably little progress has been made in reducing the impact even with judicious recruitment strategies. We hope that our data-driven stratified sampling methodology might be applied elsewhere to enable future studies to better represent their target population, improve equity, diversity and inclusion of trial participants, and account for healthy volunteer effects.
